# Out of pocket expenditure and distress financing on cesarean delivery in India: evidence from NFHS-5

**DOI:** 10.1186/s12913-023-09980-w

**Published:** 2023-09-07

**Authors:** Rajeev Ranjan Singh, Anjali Sharma, Sanjay K. Mohanty

**Affiliations:** 1 International Institute for Population Sciences, Mumbai, 400088 India; 2 Department of Population and Development, International Institute for Population Sciences, Mumbai, 400088 India

**Keywords:** Cesarean delivery, Out-of-pocket expenditure, Distress financing, India, NFHS

## Abstract

**Background:**

Though over three-fourths of all births receive medical attention in India, the rate of cesarean delivery (22%) is twice higher than the WHO recommended level. Cesarean deliveries entail high costs and may lead to financial catastrophe for households. This paper examines the out-of-pocket expenditure (OOPE) and distress financing of cesarean deliveries in India.

**Methods:**

We used data from the latest round of the National Family Health Survey conducted during 2019–21. The survey covered 636,699 households, and 724,115 women in the age group 15–49 years. We have used 159,643 births those delivered three years preceding the survey for whom the question on cost was canvassed. Descriptive analysis, bivariate analysis, concentration index (CI), and concentration curve (CC) were used in the analysis.

**Result:**

Cesarean deliveries in India was estimated at 14.08%, in private health centres and 9.96%  in public health centres. The prevalence of cesarean delivery increases with age, educational attainment, wealth quintile, BMI and high for those who had pregnancy complications, and previous birth as cesarean. The OOPE on cesarean births was US$133. It was US$498 in private health centres and US$99 in public health centres. The extent of distress financing of any cesarean delivery was 15.37%; 27% for those who delivered in private health centres compared to 16.61% for those who delivered in public health centres. The odds of financial distress arising due to OOPE on cesarean delivery increased with the increase of OOPE [AOR:10.00, 95% CI, 9.35–10.70]. Distress financing increased with birth order and was higher among those with low education and those who belonged to lower socioeconomic strata.

**Conclusion:**

High OOPE on a cesarean delivery leads to distress financing in India. Timely monitoring of pregnancy and providing comprehensive pregnancy care, improving the quality of primary health centres to conduct cesarean deliveries, and regulating private health centres may reduce the high OOPE and financial distress due to cesarean deliveries in India.

**Supplementary Information:**

The online version contains supplementary material available at 10.1186/s12913-023-09980-w.

## Introduction

Universal health coverage (UHC) has two components; access to quality healthcare services and providing financial protection to the population. High out of pocket expenditure (OOPE) often leads to catastrophic health spending and impoverishment [1, 2]. As a result, it is associated with limited or no use of health care, which leads to untreated morbidity, and increased risk to the lives of poor mothers. Globally, countries are converging on the issue of narrowing maternal mortality, increasing child survival, and improving access to essential maternal care but are experiencing an increasing number of cesarean deliveries and rising catastrophic health spending [3, 4]. Reducing maternal and childhood mortality, improving access to quality reproductive health services, and achieving universal health coverage are critical strategies to achieve the health-related Sustainable Development Goals (SDGs).

Cesarean section is a surgical procedure that prevents maternal and newborn mortality and pregnancy complications when used for medically indicated reasons. The WHO threshold of 10% of cesarean deliveries is essential for saving the lives of mothers and newborns [5],). Globally, cesarean deliveries have increased from 7% in 1990 to 21% by 2018 [6]. Reasons for the rising trend of cesarean delivery are,biological (foetal distress, arrest of descent, multiple gestations, and foetal indications), behavioural (fear and anxiety of labour pain, risk of pelvic trauma, and concern about the safety of child), and socio-demographic (age, educational attainment, wealth status, body mass index, and previous cesarean delivery) [7–10]. An observational cohort study of three decades has identified that 56% of the increase in cesarean deliveries is attributable to maternal age, body mass index, parity, and history of previous births as cesarean, and 10% is attributed to obstetrical management of high-risk pregnancies, multiple gestations, malpresentation, and preterm singleton birth [11]. Studies have found that the probabilities of cesarean delivery are higher in private health facilities [10, 12] and, hence, there is a higher utilisation among the wealthier population [13, 14].

Since the implementation of the National Health Mission (NHM) in 2005, institutional delivery in India has increased by twofold, from 46% in 2005–06 to 89.4% by 2019–21 [15] and the socioeconomic and regional inequality has reduced [16]. At the same time, cesarean deliveries have increased from 8.5% in 2005–06 to 22% in 2019–21, which has increased health spending [15]. In 2019–21, the average cost of delivery in any health facility in India was ₹10,035 compared to ₹24,663 in private health facilities. The Janani Suraksha Yojana (JSY) under NHM provides a financial incentive for delivery in accredited private or public health centres to increase institutional delivery and reduce OOPE. The financial incentive for institutional delivery varies across states as some state governments make additional provisions. In 2022, the stipulated amount in the low performing states (Uttar Pradesh, Uttarakhand, Bihar, Jharkhand, Madhya Pradesh, Chhattisgarh, Himachal Pradesh, Rajasthan, Odisha, and Jammu & Kashmir) was ₹2000 (₹1400 for mother and ₹600 for Asha worker) in rural areas, and ₹1400 (₹1000 for mother and ₹400 for Asha worker) in urban areas, whereas in the high performing states the amount was ₹1300 (₹700 for mother and ₹600 for Asha worker) in rural areas and ₹1000 (₹600 for mother and ₹400 for Asha worker) in urban areas. In the case of cesarean deliveries, financial assistance of up to ₹1,500 per pregnant woman is given to hire a private expert for performing the surgery in the absence of government-employed medical specialists [17].

The increase in cesarean deliveries across states and socioeconomic groups has aggravated the financial burden. The provisioning of services in public health centres is limited, whereas private insurance often excludes delivery services from its ambit. A number of studies have estimated the OOPE on institutional deliveries across socioeconomic groups and states of India [18–21]. A recent study has estimated the socioeconomic variations of OOPE and distress financing of institutional deliveries by healthcare providers in India [20]. Findings from these studies suggest that the OOPE and the extent of distress financing of institutional delivery were higher in the case of deliveries conducted in private health centers and in the poorer states of India as well as among poor mothers characterised by a low level of education, underweight, a previous cesarean delivery, and complicated delivery. The NHM has increased institutional deliveries but has been ineffective in reducing poor households' financial burden [22, 23]. While the earlier studies have estimated the OOPE and distress financing of institutional delivery in general, we provide these estimates for cesarean deliveries specially and examine the socioeconomic inequality in the distress financing of cesarean deliveries since such deliveries have been increasing over time, expensive and largely carried out at private health centers. Further, our estimates are based on the most recent data (NFHS 5, 2019–21), while previous studies were based on the older rounds of NFHS. Understanding the nuances of OOPE and distress financing on cesarean deliveries in India is crucial for developing targeted and effective policies to ensure financial protection from high costs and improve maternal health outcomes.

## Data and methods

The study used data from the latest round of the National Family Health Survey (NFHS-5) conducted during 2019–21. NFHS-5 is the fifth round of the Demographic and Health Survey (DHS) in India, which primarily collects individual and household demographic and health data. The survey was conducted under the aegis of the Ministry of Health and Family Welfare (MoHFW) with technical support from ICF. The survey used multilevel stratified sampling, using the 2011 Census as the sampling frame for selecting the Primary Sampling Units (PSUs). In the case of rural areas, villages were considered PSUs, whereas, in urban areas, Census Enumeration Blocks (CEBs) were used as PSUs. The survey covered 636,699 households, comprising 724,115 women in the age group 15–49 years and 101,839 men in the age group 15–54 years across all the states and union territories (UTs) of India. The sample design, methodology, and findings are available in the national report [15].

In addition to demographic and health information, the NFHS-5 collected data on OOPE on institutional deliveries and sources to meet the OOPE. There was a total of 176,843 last births to mothers during five years preceding the survey, of which 159,643 deliveries were conducted at a health centre. The data on OOPE was edited for errors that arise due to the numerical value of “do not know” or “missing” data. The survey was conducted in two phases over two years; the reference year for OOPE was not uniform. The estimates were adjusted at 2021 prices using a price deflator^1^ for comparable OOPE estimates. We adjusted the price to a constant price based on the monthly consumer price index (CPI) value and the month of childbirth. We have presented the OOPE in US$ with an equivalence of US$1 = ₹73.78, the average exchange rate during the 2019, 2020, and 2021 survey periods. A question on the source of finance for the delivery was asked for each birth. The sources were categorised as; only savings, selling assets & borrowing money, saving along with selling assets & borrowing money, insurance & others. All institution deliveries, whether cesarean or non-cesarean, that took place in private and public health facilities were combined based on which four comparator groups were formed, that is, private-cesarean, private non-cesarean, public-cesarean, and public non-cesarean.

## Methodology

Descriptive analysis, bivariate analysis, concentration index (CI), and concentration curve (CC) were used in the analysis. The analysis was carried out in three stages. In the first stage, we examined the variations in cesarean and non-cesarean deliveries between public and private health facilities and estimated state-specific OOPE and distress financing. In the second stage, we estimated the concentration indices and concentration curves to examine the inequality in cesarean delivery. In the third stage, we carried out logistic regression to examine the determinant of distress financing.

## Outcome variables

Three outcome variables were estimated. a) distribution of cesarean and non-cesarean deliveries at public and private health centres, b) OOPE, and c) distress financing. OOPE was calculated for the last birth and estimated at 2021 prices. Distress financing was defined if OOPE on cesarean delivery was met by borrowing money or selling assets or by utilising savings along with borrowing money or selling assets. The outcome variables were coded dichotomously as ‘0’ if the reply was No and ‘1’ if the reply was Yes.

## Independent variables

The independent variables used were mother’s age (grouped as 15–24 years, 25–34 years, 35 + years), sex of child (male/female), mother’s level of education (no education, primary, secondary, higher secondary and above), birth order (1/2/3/4 +), place of residence (urban/rural), wealth quintile (poorest, poorer, middle, richer, richest), BMI (underweight, normal, overweight), pregnancy complications (no/yes), and repeat cesarean delivery (no/yes).

## Estimating concentration curve and concentration index

We used the concentration curve (CC) to plot the cumulative proportion of the population based on wealth against the cumulative population using cesarean delivery care services in health facilities (public or private). A CC below the line of equality shows a pro-rich use of services, while a CC above the line of inequality shows a pro-poor use of services. The concentration index (CI) is a numerical inequality estimate and ranges from -1 to + 1, with ‘0’ representing uniform distribution [24].

## Results

Table 1 shows the descriptive characteristics of cesarean and non-cesarean deliveries in private and public health centres. The mean age of mothers was 27 years, and the mean years of schooling was eight years. The mean unadjusted OOPE for institutional delivery was US$121. The cost of institutional delivery of about 15.37% of births was met through distress financing, of which the cost of around 9% of births was met through selling assets & borrowing money, and that of 6.40% of births was met by selling assets and borrowing money along with savings. Among mothers who availed of cesarean delivery services in private health centres, the mean age was 28 years, and the mean years of schooling were 11 years; only 18% of them belonged to the poorest and poorer wealth quintiles. The mean unadjusted OOPE was US$446, and the number of surviving children was 1.64. The extent of distress financing for cesarean deliveries in private health centres was 27%, of which 11.8% was through selling assets & borrowing money and 15.9% by using savings along with selling assets & borrowing money.

**Table 1:** Descriptive statistics of sample population for cesarean and Non- cesarean delivery, India, 2019–21

Background characteristics	Private and Cesarean	Private and Non-Cesarean	Public and cesarean	Public and Non-cesarean	Total
Mean age of mother	28	27	27	27	27
Mean years of schooling of mother	11	10	9	9	8
Percent urban	44	41	36	37	28
Mean number of surviving children	1.64	1.93	1.75	1.68	2.07
Percentage of women belonging to poorest and poorer wealth quintile	18	22	31	31	44
Percent SC/ST	20	22	32	32	33
Mean unadjusted OOPE	446	217	78	91	121
**Distress Financing (%)**	27.09	19.3	16.61	13.76	15.37
Only Savings	64.2	70.87	64.69	62.11	57.88
Selling and Borrowing	11.18	9.38	10.05	9.72	8.96
Insurance & others	4.5	2.65	3.65	3.06	2.96
Saving along with selling & borrowing	15.9	9.92	6.56	4.04	6.4
did not pay	4.21	7.18	15.05	21.08	23.79
Number of women (N)	24,785	25,454	20,641	17,619	159,643

Table 2 shows the state-wise variations in cesarean and non-cesarean deliveries in public and private health centres. The rate of cesarean births varied enormously across states and was higher in private health centres. It was the highest in Telangana (39%), followed by Andhra Pradesh (30%) and Kerala (27%), and the lowest in Arunachala Pradesh (2%). The states/UTs with the highest rate of cesarean deliveries in public health centres were Jammu & Kashmir (39%), followed by Puducherry (27%), and Chandigarh (25%). Similarly, (Supplementary Table 1) shows the distribution of cesarean and non-cesarean deliveries by types of health centres and background characteristics.

**Table 2:** Percent share of cesarean and non-cesarean delivery by type of providers across states, India, 2019–21

State^a^	Private and cesarean	Private and non-cesarean	Public and cesarean	Public and non-cesarean	Total Institutional Delivery	Delivery at home	N
**India**	**14.08**	**14.39**	**9.96**	**51.90**	**90.27**	**9.73**	**176,843**
Telangana	38.82	8.46	24.09	26.44	97.81	2.19	5,429
Andhra Pradesh	30.23	16.29	14.84	36	97.37	2.63	2,092
Kerala	26.46	39.1	13.06	21.27	99.89	0.11	2,360
Punjab	24.39	18.52	16.39	35.92	95.22	4.78	4,520
Tamil Nadu	21.91	12.24	25.47	40.09	99.71	0.29	5,228
Goa	21.74	22.25	17.47	38.23	99.69	0.31	322
Karnataka	18.26	15.76	15.69	48.23	97.94	2.06	6,389
Gujarat	17.63	35.19	5.85	36.77	95.44	4.56	7,575
West Bengal	17.31	4.01	17.52	54.37	93.21	6.79	4,894
Maharashtra	16.51	24.22	10.91	43.92	95.56	4.44	7,415
Daman & Diu	14.57	22.02	11.26	49.07	96.92	3.08	368
Haryana	14.11	25.89	7.65	48.67	96.32	3.68	5,162
Uttarakhand	14	18.7	7.77	44.59	85.06	14.94	2,966
Delhi	13.97	17.2	11.46	50.39	93.02	6.98	2,379
Lakshadweep	13.01	18.93	18.97	49.09	100	0	251
Manipur	12.19	9.48	16.59	44.51	82.77	17.23	2,511
Uttar Pradesh	11.56	15.89	4.04	53.86	85.35	14.65	25,556
Odisha	10.6	4.54	12.99	65.19	93.32	6.68	7,141
Dadar & Nagar Haveli	10.43	11.97	14.08	60.1	96.58	3.42	267
Andaman & Nicobar Island	10.38	2.91	20.76	65.45	99.50	0.5	401
Chhattisgarh	9.95	7.16	6.8	63.18	87.09	12.91	6,526
Jharkhand	9.76	10.33	4.5	52.16	76.75	23.25	7,465
Puducherry	9.48	14.19	27.04	49.17	99.88	0.12	616
Himachal Pradesh	9.41	8.98	13.23	58.33	89.5	10.05	2,145
Bihar	9.02	12.2	2.33	54.18	77.73	22.27	13,874
Sikkim	8.74	7.83	24.14	54.47	95.18	4.82	569
Tripura	7.78	3.37	18.89	60.59	90.63	9.37	1,860
Assam	7.51	3.31	12.07	63.25	86.14	13.86	9,247
Chandigarh	6.43	6.85	25.07	59.35	97.69	2.31	144
Madhya Pradesh	6.35	5.56	7.45	72.54	91.90	8.1	11,700
Rajasthan	5.48	13.36	6.28	70.69	95.81	4.19	10,831
Jammu & Kashmir	4.76	1.09	38.46	48.86	93.16	6.84	5,367
Meghalaya	4.59	6.63	4.88	48.13	64.23	35.77	4,602
Mizoram	3.91	9.05	8.2	65.73	86.88	13.12	1,896
Nagaland	2.94	7.61	3.58	34.2	48.33	51.67	2,205
Arunachal Pradesh	2.19	2.33	13.63	63.09	81.24	18.76	4,570

^a^states are sorted in descending order by cesarean in private health centers

**Fig. 1:**
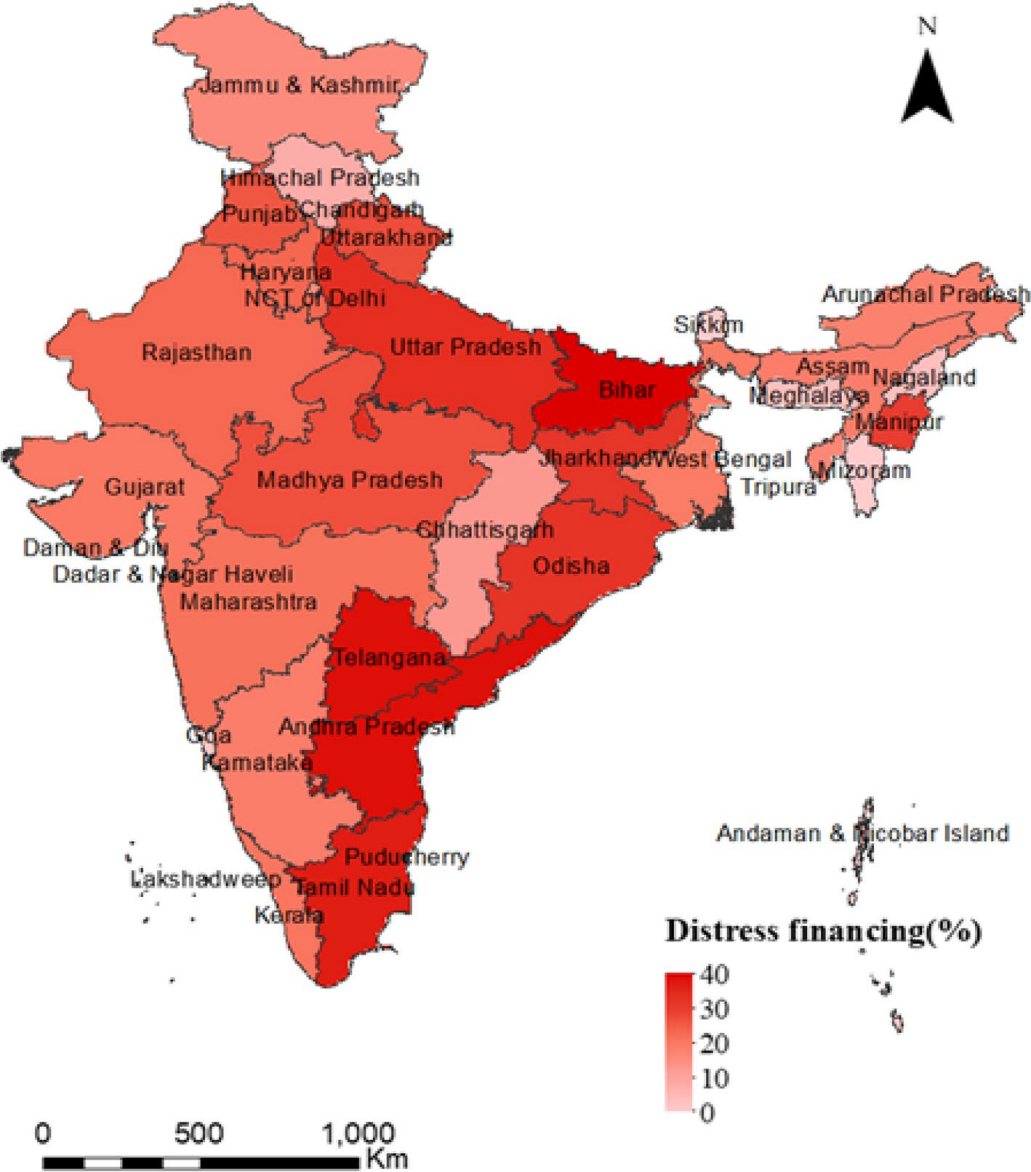
Mean OOPE (US$) for cesarean delivery in private health facilities across states, India, 2019–21

**Fig. 2:**
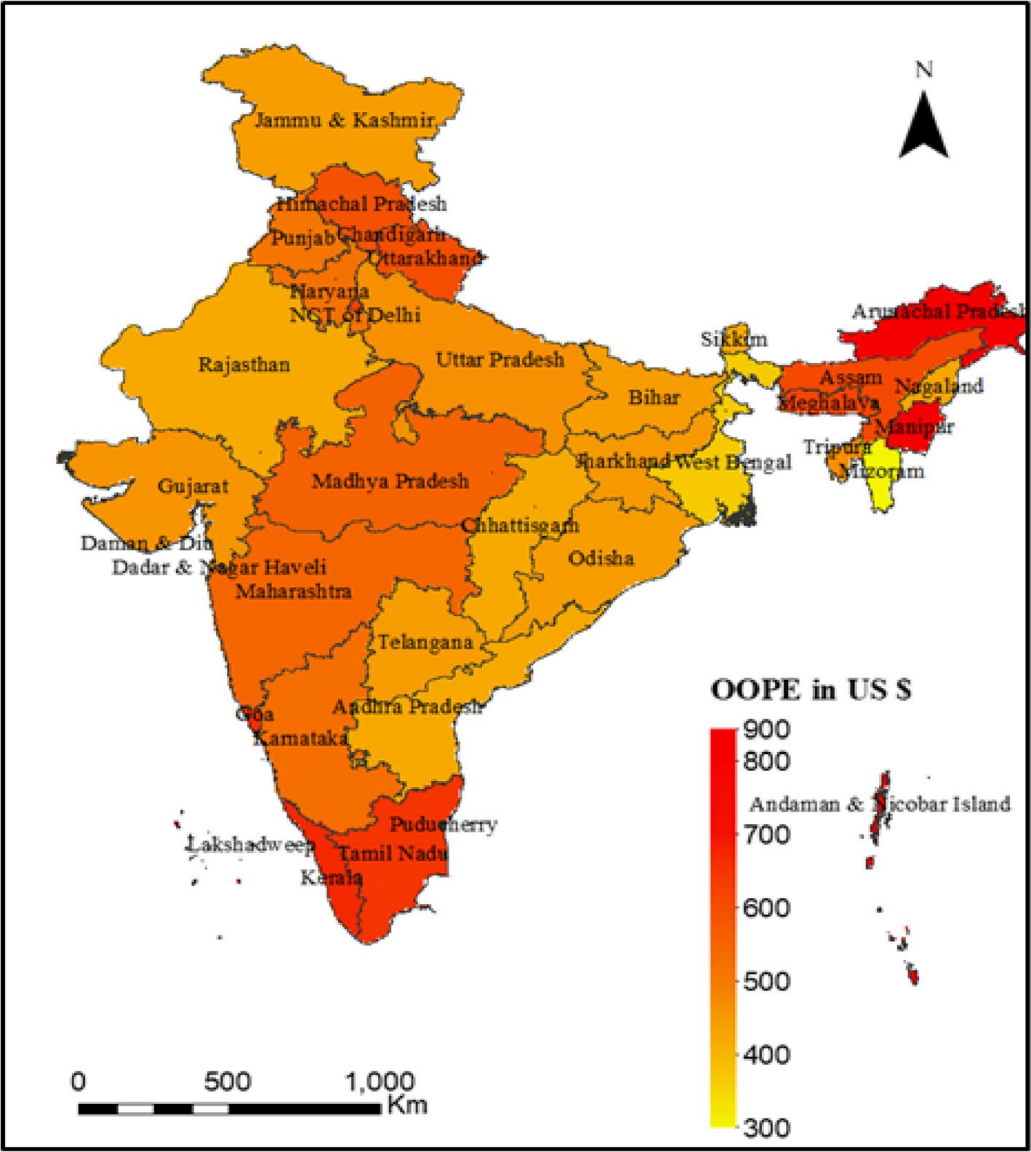
Percentage of births that met the OOPE by distress financing for cesarean deliveries in states of India, 2019–21

Figure 1: presents the mean OOPE for cesarean delivery in private health centres across the states of India. The overall mean OOPE in India was US$496, ranging from US$305 to US$848 across the states. The mean OOPE was highest in Manipur (US$848), followed by Arunachal Pradesh (US$717) and Kerala (US$679), and it was lowest in Mizoram (US$305), followed by West Bengal (US$370) and Andhra Pradesh (US$422). Figure 2 presents the percentage of distress financing for cesarean delivery across states. At the national level, distress financing was estimated at 15.37%. The distress financing was highest in Bihar, followed by Andhra Pradesh and Telangana, and it was lowest in Mizoram, followed by Sikkim and Goa.

Table 3 shows the mean OOPE for cesarean and non-cesarean deliveries in public and private health centres across India and its states. At the national level, the overall mean OOPE was US$132. For cesarean deliveries, the mean OOPE was US$496 in private health centres and US$99 in public health centres. On the other hand, the mean OOPE was US$239 for non-cesarean deliveries in private health centres and US$35 for public health centres. The overall mean OOPE was highest in Kerala (US$378), followed by Manipur (US$308) and Goa (US$278), and lowest in Meghalaya (US$58), followed by Nagaland (US$62) and Madhya Pradesh (US$72). With regards to cesarean deliveries in private health centres, the mean OOPE was highest in Manipur (US$848), followed by Arunachal Pradesh (US$717) and Chandigarh (US$679), and it was lowest in Mizoram (US$305) followed by West Bengal (US$370) and Andhra Pradesh (US$422). In the case of cesarean deliveries in public health centres, the mean OOPE was highest in Manipur (US$430), followed by Arunachal Pradesh (US$265) and Nagaland (US$257). It was lowest in Dadra and Nagar Haveli (US$13), followed by Daman and Diu (US$ 40) and Puducherry (US$52). Similarly, (Supplementary Table 2) shows the mean OOPE (in US$) for cesarean and non-cesarean deliveries by types of health centres and background characteristics.

**Table 3:** Mean OOPE (in US$) for cesarean and non-cesarean delivery at 2021 prices by type of providers across states, India, 2019–21

**State**^b^	**OOPE at 2021 prices (in US$**^a^**)**				
	**Private and Cesarean**	**Private and Non-Cesarean**	**Public and cesarean**	**Public and Non-cesarean**	**Total**
**India**	**496**	**239**	**99**	**35**	**132**
Manipur	848	404	430	213	308
Andaman & Nicobar	833	487	53	41	138
Lakshadweep	787	517	61	33	226
Arunachal Pradesh	717	326	265	109	128
Chandigarh	679	573	119	48	141
Kerala	675	429	113	80	378
Goa	669	494	78	53	289
Tamil Nadu	653	437	67	47	232
Delhi	626	381	77	31	176
Meghalaya	622	176	94	28	58
Assam	616	245	187	66	119
Uttarakhand	600	299	123	37	166
Himachal Pradesh	594	307	81	47	121
Madhya Pradesh	563	268	89	20	72
Maharashtra	553	282	88	39	186
Karnataka	530	306	135	59	195
Haryana	522	226	47	21	146
Punjab	510	246	96	41	200
Dadar & Nagar Haveli	505	169	13	7	79
Daman & Diu	474	142	40	15	112
Puducherry	471	339	52	57	135
Tripura	471	198	154	65	112
Uttar Pradesh	464	177	128	28	102
Gujarat	461	166	74	20	151
Jharkhand	453	184	103	25	81
Bihar	444	165	149	36	83
Orissa	444	203	147	47	106
Telangana	443	255	79	55	227
Jammu & Kashmir	443	255	112	74	103
Nagaland	435	245	257	62	62
Chhattisgarh	428	218	113	22	79
Rajasthan	427	171	74	26	69
Sikkim	426	193	173	85	141
Andhra Pradesh	422	241	65	35	189
West Bengal	370	196	79	30	102
Mizoram	305	199	164	42	71

^a^1 dollar = 73.78 INR (average of 2019, 2020 and 2021 exchange prices as the survey was done during the period)

^b^states are sorted in descending order by cesarean delivery in private health centers

**Fig. 3:**
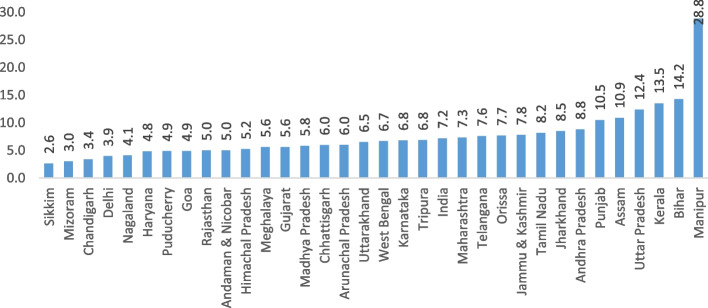
OOPE on institutional delivery as a share of per capita state domestic product across the states of India, 2019–21

Figure 3 presents the OOPE on institutional delivery as a share of per capita state domestic product (SDPP), a barometer of the economic progress of a state. The national average OOPE on institutional delivery as a share of SDPP was 7.2%. Across the states, it was highest in Manipur (28.8%), followed by Bihar (14.2%), Kerala (13.5%), and Uttar Pradesh (12.4%). In contrast, it was lowest in Chandigarh (3.4%), Delhi (3.9%), and Haryana (4.8%) (Supplementary Table 3).

**Fig. 4:**
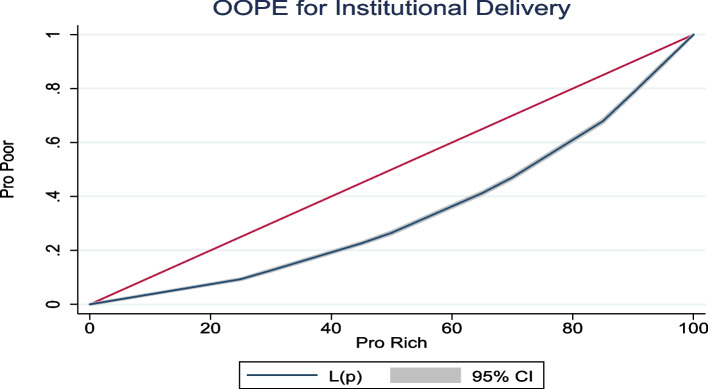
Concentration curve of OOPE for institutional delivery in India, 2019–21

Figure 4 presents the concentration curve of OOPE for institutional delivery. The concentration curve was below the line of inequality, implying a higher concentration among wealthy people. The concentration index was (0.327, 95% CI: 0.322–0.331).

**Fig. 5:**
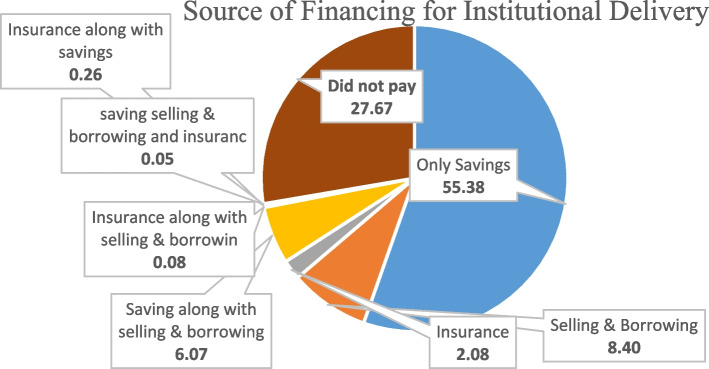
Percent Distribution of Sources of OOPE financing for institutional deliveries in India, 2019–21

Figure 5 presents the distribution of the sources of OOPE financing for institutional delivery. The OOPE on institutional deliveries was largely met by using savings (55.38%) followed by selling assets & borrowing money (8.40%), and by using savings along with selling assets & borrowing money (6.07%). About 27.67% of mothers did not pay anything to avail of the service (Supplementary Table 4).

Table 4: shows the mean OOPE for cesarean deliveries by the source of financing across the states. At the national level, the mean OOPE on cesarean deliveries was the lowest among those who used only their savings to finance the delivery (US$157), followed by those who financed the procedure by selling assets and borrowing money (US$176). The mean OOPE on cesarean deliveries was highest among those who met the expenditure by using their savings along with selling assets and borrowing money (US$303). The mean OOPE was US$229 among those who had to resort to any distress financing. The mean OOPE for cesarean deliveries financed by using savings along with selling assets and borrowing money was highest in Kerala (US$613), followed by Dadra and Nagar Haveli (US$589) and Delhi (US$538). It was lowest in Andaman and Nicobar (US$89), followed by Mizoram (US$157) and Jammu & Kashmir (US$162). Similarly, the mean OOPE for cesarean deliveries through any form of distress financing across the states was highest in Dadra and Nagar Haveli (US$608), followed by Kerala (US$550) and Manipur (US$412). It was lowest in Andaman& Nicobar (US$35), followed by Daman & Diu (US$65) and Jammu & Kashmir (US$134). (Supplementary Table 5) shows the percentage of births that were funded by any distress financing and the percent distribution of source of financing for cesarean deliveries by background characteristics.

**Table 4:** Mean OOPE (in US$) for cesarean delivery by source of financing across states, India, 2019–21

State	Savings only	Selling and borrowing only	Saving along with selling and borrowing	Insurance and others	Any distress
**India**	**157**	**176**	**303**	**188**	**229**
Dadar & Nagar Haveli	139	683	589	32	608
Kerala	426	487	613	456	550
Manipur	356	334	461	488	412
Delhi	236	245	538	293	379
Tamil Nadu	199	317	503	448	377
Himachal Pradesh	188	233	571	129	367
Uttarakhand	217	317	342	352	330
Punjab	238	265	411	152	315
Haryana	192	259	357	170	313
Telangana	216	272	377	303	309
Chandigarh	176	319	254	854	301
Andhra Pradesh	187	266	348	194	300
Lakshadweep	356	165	391	0	280
Karnataka	207	226	328	302	266
Sikkim	188	265	238	268	260
Maharashtra	219	177	323	293	232
Arunachal Pradesh	188	257	214	358	231
Goa	306	155	361	433	228
Uttar Pradesh	117	130	299	161	225
Gujarat	195	160	320	130	209
Jharkhand	108	108	335	141	189
Puducherry	143	141	297	169	183
Nagaland	150	117	259	151	180
Tripura	154	154	212	231	179
Orissa	111	119	242	228	172
Chhattisgarh	117	105	320	125	168
West Bengal	142	122	244	83	166
Bihar	97	126	209	103	159
Madhya Pradesh	104	108	301	143	157
Assam	161	101	194	157	154
Meghalaya	167	144	191	149	152
Mizoram	162	132	157	239	148
Rajasthan	83	110	181	129	140
Jammu & Kashmir	115	109	162	166	134
Daman & Diu	160	10	233	53	65
Andaman & Nicobar	233	31	89	161	35

- $1 = 73.78INR (average of 2019, 2020 and 2021 exchange prices as the survey was done during the period)

- states are sorted in descending order by any distress financing

- ^a^‘Insurance along with savings (463), ‘saving, selling & borrowing and insurance (87) and ‘Insurance along with selling & borrowing (136)’, are categorized under any insurance & others.

Table 5 shows the concentration index by the source of financing for cesarean deliveries across the states of India. At the national level, the concentration index for financing cesarean deliveries through savings was pro-rich [ 0.043, 95% CI: 0.041, 0.045]. On the other hand, the concentration index for meeting OOPE by selling assets & borrowing money was pro-poor [-0.167 95% CI: -0.174, -0.159], as was the concentration index for overall distress financing was also pro-poor [-0.166 95% CI: -0.173, -0.158]. The concentration index for financing cesarean deliveries through savings was pro-rich across all the states except Mizoram (-0.025) and Goa (-0.005). The concertation index was highest in Gujarat [0.084 95% CI: 0.076, 0.093], followed by Nagaland [0.079 95% CI: 0.046, 0.113] and Bihar [0.073 95% CI: 0.064, 0.083], whereas it was lowest in Sikkim [0.001 95% CI: -0.036, 0.039] followed by Jharkhand [0.003 95% CI: 0.002, 0.004] and Madhya Pradesh [ 0.005 95% CI: 0.038, 0.062]. Conversely, the concentration index to meet OOPE on cesarean deliveries by selling assets and borrowing money was pro-poor across all the states except Chandigarh (0.142). It was highest in Andaman & Nicobar [-0.872 95% CI: -1.666, -0.078], followed by Sikkim [-0.670 95% CI: -0.977, -0.363] and Mizoram [-0.521 95% CI: -0.761, -0.281], and it was lowest in Tamil Nadu [-0.063 95% CI: -0.085, -0.041] followed by Andhra Pradesh [ -0.067 95% CI: -0.094, -0.040] and Daman & Diu [-0.067 95% CI: -0.094, -0.040].

**Table 5:** concentration Index by source of financing for cesarean delivery across states, India, 2019–21

State	Only Savings		Only Selling Asset or Borrowing		Any Distress	
	**Index Value**	**Confidence Interval**	**Index Value**	**Confidence Interval**	**Index Value**	**Confidence Interval**
**India**	**0.043**	**(0.041, 0.045)**	**-0.167**	**(-0.174, -0.159)**	**-0.166**	**(-0.173, -0.158)**
Andaman & Nicobar Island	0.048	(0.004, 0.092)	-0.872	(-1.666, -0.078)	-0.872	(-1.666, -0.078)
Sikkim	0.001	(-0.036, 0.039)	-0.67	(-0.977, -0.363)	-0.587	(-0.888, -0.287)
Mizoram	-0.025	(-0.071, 0.021)	-0.521	(-0.761, -0.281)	-0.521	(-0.761, -0.281)
Nagaland	0.079	(0.046, 0.113)	-0.489	(-0.637, -0.341)	-0.48	(-0.632, -0.328)
Lakshadweep	0.023	(-0.013, 0.058)	-0.46	(-0.749, -0.171)	-0.46	(-0.749, -0.171)
Chhattisgarh	0.027	(0.015, 0.039)	-0.386	(-0.445, -0.328)	-0.367	(-0.427, -0.308)
Dadar & Nagar Haveli	0.061	(0.017, 0.104)	-0.284	(-0.659, 0.090)	-0.284	(-0.659, 0.090)
Assam	0.024	(0.016, 0.032)	-0.279	(-0.305, -0.253)	-0.28	(-0.306, -0.253)
Gujarat	0.084	(0.076, 0.093)	-0.268	(-0.303, -0.233)	-0.267	(-0.302, -0.232)
Manipur	0.064	(0.051, 0.076)	-0.261	(-0.295, -0.227)	-0.263	(-0.297, -0.228)
Arunachal Pradesh	0.018	(0.006, 0.031)	-0.254	(-0.334, -0.173)	-0.254	(-0.334, -0.173)
Goa	-0.005	(-0.025, 0.015)	-0.254	(-0.496, -0.011)	-0.254	(-0.496, -0.011)
Meghalaya	0.073	(0.031, 0.115)	-0.225	(-0.311, -0.140)	-0.234	(-0.326, -0.142)
Uttarakhand	0.034	(0.02, 0.047)	-0.222	(-0.280, -0.164)	-0.228	(-0.287, -0.169)
Karnataka	0.036	(0.029, 0.043)	-0.224	(-0.252, -0.196)	-0.22	(-0.249, -0.192)
Jammu & Kashmir	0.032	(0.025, 0.039)	-0.219	(-0.247, -0.190)	-0.219	(-0.247, -0.190)
Delhi	0.018	(0.007, 0.03)	-0.23	(-0.297, -0.162)	-0.219	(-0.287, -0.151)
Haryana	0.044	(0.034, 0.053)	-0.214	(-0.254, -0.175)	-0.217	(-0.256, -0.177)
Orissa	0.043	(0.036, 0.051)	-0.214	(-0.239, -0.189)	-0.212	(-0.237, -0.187)
Tripura	0.049	(0.029, 0.069)	-0.207	(-0.292, -0.122)	-0.204	(-0.29, -0.118)
Bihar	0.073	(0.064, 0.083)	-0.201	(-0.221, -0.181)	-0.202	(-0.222, -0.182)
West Bengal	0.068	(0.059, 0.076)	-0.204	(-0.238, -0.169)	-0.2	(-0.235, -0.166)
Punjab	0.066	(0.057, 0.074)	-0.195	(-0.223, -0.168)	-0.195	(-0.223, -0.168)
Himachal Pradesh	0.026	(0.008, 0.044)	-0.234	(-0.347, -0.120)	-0.179	(-0.283, -0.075)
Kerala	0.054	(0.042, 0.065)	-0.172	(-0.213, -0.131)	-0.175	(-0.216, -0.134)
Rajasthan	0.047	(0.038, 0.056)	-0.153	(-0.193, -0.114)	-0.154	(-0.194, -0.114)
Telangana	0.073	(0.066, 0.08)	-0.139	(-0.156, -0.121)	-0.142	(-0.160, -0.125)
Uttar Pradesh	0.029	(0.024, 0.033)	-0.14	(-0.156, -0.124)	-0.14	(-0.157, -0.124)
Maharashtra	0.024	(0.015, 0.033)	-0.136	(-0.181, -0.091)	-0.139	(-0.185, -0.094)
Jharkhand	0.03	(0.02, 0.04)	-0.137	(-0.168, -0.105)	-0.131	(-0.163, -0.099)
Madhya Pradesh	0.05	(0.038, 0.062)	-0.116	(-0.150, -0.083)	-0.108	(-0.142, -0.074)
Puducherry	0.044	(0.011, 0.077)	-0.082	(-0.161, -0.003)	-0.082	(-0.161, -0.003)
Daman & Diu	-0.018	(-0.044, 0.008)	-0.067	(-0.151, 0.016)	-0.067	(-0.151, 0.016)
Tamil Nadu	0.044	(0.036, 0.052)	-0.063	(-0.085, -0.041)	-0.065	(-0.087, -0.042)
Andhra Pradesh	0.038	(0.024, 0.052)	-0.067	(-0.094, -0.040)	-0.059	(-0.087, -0.031)
Chandigarh	0.025	(-0.009, 0.058)	0.142	(0.037, 0.247)	0.142	(0.037, 0.247)

^*^States are sorted in descending order of any distress financing

**Fig. 6:**
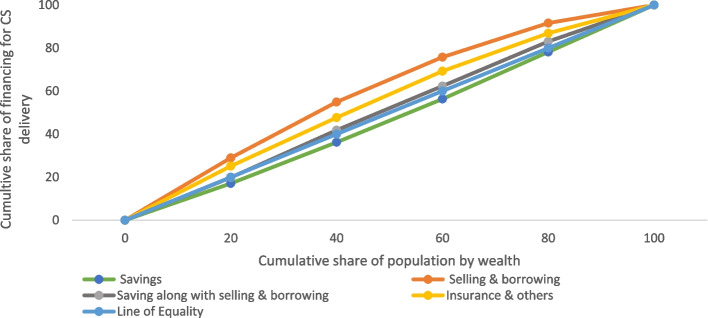
Concentration Curve for different sources of financing for cesarean delivery, India, 2019–21

Figure 6 shows the concentration curve by the sources of financing for cesarean deliveries. The sources like selling assets & borrowing money, using insurance & others, and using savings, along with selling assets & borrowing money, were above the line of equality, implying a higher concentration among people with low incomes. The selling assets & borrowing money line was furthest from the line of equality, indicating a deeper concentration among people with low incomes. Further, savings as the only financing source was below the equality line, implying a higher concentration among richer.

Table 6 shows the odds of distress financing for cesarean deliveries by socioeconomic variables. The likelihood of distress financing increased with an increase in the mean OOPE. The odds of distress financing were 10.00 [AOR: 10.00 95% CI: 9.35 -10.70] times higher among those whose mean OOPE was US$271 and above compared to those who spent up to US$14 to avail of cesarean services. Among female-headed households, the odds of distress financing were 1.03 [AOR:1.03 95% CI: 0.99–1.08] times higher than male-headed households. The odds of distress financing were 1.25 [AOR: 1.25 95% CI: 1.18–1.33] times higher for cesarean deliveries in private health centres than non-cesarean deliveries in public health centres. The odds of distress financing were 1.16 [AOR: 1.16 95% CI: 1.09–1.23] times higher among mothers in the age group 15–24 years compared to mothers in the 25–35 age group. By level of education, the likelihood of distress financing was 1.63 [AOR: 1.63 95% CI: 1.54–1.71] times higher among mothers with no education and 1.70 [AOR: 1.70 95% CI: 1.61–1.80] time higher among mother having primary education compared to mothers with higher secondary and above-education. Similarly, the odds of distress financing were 3.95 [AOR: 3.95 95% CI: 3.68–4.24] times higher among the poorest and 3.03 [AOR: 3.03 95% CI: 2.84–3.23] time higher among poorer compare to the richest wealth quantile. Further, the odds of distress financing were 1.05 [AOR: 1.05 95% CI: 1.01–1.09] times higher among underweight mothers than normal-weight mothers.

**Table 6:** Odds of distress financing for cesarean delivery by socioeconomic variables in India, 2019–21

Variables	Odds Ratio	95% confidence interval
**OOPE at Constant Price (in US$)**		
0 -14	1.00	
14—68	3.32***	(3.16, 3.48)
68—136	5.60***	(5.30, 5.93)
136—203	7.02***	(6.56, 7.51)
203—271	7.61***	(7.01, 8.27)
271 USD +	10.00***	(9.35, 10.70)
**Type of Delivery**		
Public Non-Cesarean	1.00	
Public Cesarean	1.07**	(1.02, 1.13)
Private Non Cesarean	0.99	(0.94, 1.05)
Private Cesarean	1.25***	(1.18, 1.33)
**Mother’s Age**		
25–34	1.00	
15–24	1.16***	(1.09, 1.23)
35 +	1.06*	(1.00, 1.11)
**Sex of Household Head**		
Male	1.00	
Female	1.03*	(0.99, 1.08)
**Sex of child**		
Female	1.00	
Male	1.004	(0.98, 1.03)
**Mother’s Education**		
Higher secondary	1.00	
No Education	1.63***	(1.54, 1.71)
Primary	1.70***	(1.61, 1.80)
Secondary	1.42***	(1.37, 1.48)
**Birth Order**		
1	1.00	
2	1.03	(0.99, 1.06)
3	1.13***	(1.07, 1.18)
4 +	1.19***	(1.13, 1.27)
**Place of Residence**		
Urban	1.00	
Rural	0.98	(0.94, 1.02)
**Wealth Quintile**		
Richest	1.00	
Poorest	3.95***	(3.68, 4.24)
Poorer	3.03***	(2.84, 3.23)
middle	2.28***	(2.14, 2.42)
richer	1.72***	(1.63, 1.83)
**Mother’s BMI**		
Normal	1.00	
Underweight	1.05*	(1.01, 1.09)
Overweight	0.95*	(0.92, 0.99)
**Pregnancy Complications**		
No	1.00	
Yes	0.96*	(0.93, 0.99)

^***^p < 0.001, **p < 0.01, *p < 0.05 (State-level Adjusted Model)

## Discussion

Despite increasing public health spending, health insurance coverage, and use of maternal services from public health centres, the OOPE, catastrophic health spending, and distress financing continue to be high in India. The increasing incidence of cesarean and institutional deliveries has been attributed to the high financial burden on the household. Using data from the most recent round of NFHS, we examined the place of delivery, type of delivery, and the sources of meeting expenditure. The salient findings of our study and plausible explanations for them are as follows.

First, the extent of cesarean deliveries and OOPE varies across socioeconomic groups and states of India. We found that higher age, higher educational attainment, higher wealth quintile, higher BMI, pregnancy complications, and a previous cesarean birth were associated with a higher rate of cesarean deliveries in India. The inter-state variations of cesarean deliveries are significant. For instance, 39% of all the deliveries in Telangana, 30% in Andhra Pradesh, and 27% in Kerala were cesarean. Second, the inflation-adjusted (at 2021 prices) OOPE of cesarean deliveries was twice higher in private health centres compared to public health centres. Among all the states in India, the OOPE on cesarean deliveries in private health centres was highest in Manipur (US$848), followed by Andaman and Nicobar (US$833) and Lakshadweep (US$787) and lowest in Mizoram (US$305). The OOPE on cesarean deliveries in public health centres was highest in Manipur (US$430), followed by Nagaland (US$257), and lowest in Dadra and Nagar Haveli (US$13). At the national level, the mean OOPE on a cesarean delivery in a private health centre was around five times higher than in a public health centre. The OOPE as a share of SDPP varies largely across states. Third, the extent of distress financing for meeting the OOPE was over 15.37% and was the highest for those who had a cesarean delivery in a private health centre (27%). Further, at the national level, the OOPE for cesarean deliveries was US$303 for those who met the expenditure by using their savings along with selling assets and borrowing money. In contrast, it was US$157 for those who met the expenditure through savings alone. The mean OOPE for distress financing was highest in Dadra and Nagar Haveli, US$608, followed by Kerala, US$550, and it was lowest in Andaman and Nicobar (US$35). Fourth, the concentration curve for the source of finance was above the line of inequality for selling assets and borrowing money, using insurance & others, and using savings along with selling assets & borrowing money, showing higher distress financing among people with low incomes. In contrast, the concertation curve for using only savings to meet OOPE was below the line of inequality, indicating higher concertation among the richer. At the national level, the concertation curve for meeting OOPE on cesarean deliveries through any form of distress financing was negatively concentrated, depicting a higher concentration among the poor. Fifth, the odds of financial distress arising from OOPE on a cesarean delivery increased with the extent of OOPE. For instance, the likelihood of incurring financial distress was significantly higher among those who spent an average of US$271 and above compared to those who spent up to US$14. The extent of distress financing increased with birth order and was also higher among mothers with low levels of education, mothers from the poor wealth quintile, and underweight mothers.

There are both clinical and non-clinical reasons behind the increasing number of cesarean deliveries in India. Mothers from higher social and economic strata demand cesarean delivery to avoid labour pain [9, 10]. Other plausible reasons may be the decreasing family size, advancements in technology, and better health facilities available at private health centres [15, 25, 26]. Similarly, the economic motive of private healthcare providers may also be one of the plausible reasons for the endorsement of cesarean delivery [10]. The unavailability of good professionals at public health centres forces people to opt for private health centres [27]. Studies suggest that the non-participation in a cash assistance program, often unintentional and caused by personal circumstances or poor geographic access, or driven by a perception of poor quality of care provided in program facilities, may lead to non-acceptance of cash-assistance programs and hence high OOPE [28, 29].

Our findings on the estimates of OOPE and distress financing of cesarean deliveries are consistent with the literature. Goli et al. [23] found that OOPE on institutional deliveries had undergone a substantial increase, which may lead to catastrophic spending by households [23]. A study by Selvaraj et al. [30] identified that a significant proportion of households spent a high share of their annual consumption expenditure on medicines and diagnostic tests, despite many free of charge government- schemes [30]. The extent of OOPE and distress financing varies across states and is higher in the poorer states of Bihar, Uttar Pradesh, and Assam. The plausible reason for variation in OOPE across states may be the variations in the provisioning of medicine, tests, user charges, and inaccessibility of the public health centres. Other reasons may be the demand for a better quality of care, the ability to afford high-paid services, and varying incentives under state-specific schemes. It may be noted that health is a state subject, and public health services are funded by state, central, and local governments. Each state's health program and priority, budget allocation, and regulation are different, which may lead to variations in the quality of services and OOPE. Besides, the level of economic development of a state also determines the ability of households to pay for and use quality services. We found that in the states of Uttar Pradesh, Manipur, Kerala, and Bihar, the share of OOPE on institutional delivery to SDPP was higher compared to the other states. In the case of Bihar and Uttar Pradesh, low income levels, poor/unavailability of public health facilities, increasing rates of cesarean delivery, and dependency on private health facilities could be the possible reasons. In Manipur, the estimates are also high, possibly due to the non-availability of facilities in the state. In the case of Kerala, the OOPE as a share of SDPP is high, possibly due to higher purchasing power parity, low fertility, and the urge for quality health facilities. It was lower in Chandigarh, Delhi, and Haryana, and the possible reasons for the availability of better public health facilities. Although NHM, JSY, and other state-specific schemes provide financial assistance to the poor, the amount stipulated is not enough to constrain OOPE and distress financing for cesarean delivery. Thus, the high OOPE forces poor mothers to resort to alternative sources of financing to meet the OOPE on cesarean delivery. It suggests that the JSY pay-offs remain low, and financial push does not provide a sufficient sum to cover the expenditure incurred on cesarean delivery.

The following are some of the limitations of the study. To begin with, we could not estimate the extent of catastrophic health spending caused by high OOPE. Further, the study could not determine the extent of cesarean deliveries were unnecessary in India. Due to data limitations, we could not quantify the extent of borrowing money and selling assets. Borrowing money at a higher interest rate may affect the household's welfare in the long run. The NFHS-5 did not collect data on the cost of borrowing and the mode of repayment. Hence, we could not account for these trade-offs in the present study. Another limitation of the study is that the OOPE reportedly incurred by mothers on cesarean delivery may have recall bias as the study has considered the most recent birth that took place during the five years preceding the survey. Other constraints may be the non-inclusion of the recent benefits offered under the Pradhan Mantri Matru Vandana Yojana (PMMVY) and Ayushman Bharat Initiative (ABY). The PMMVY was launched in 2017, while ABY was launched in 2018. The ABY launched in September 2018, and the NFHS-5 survey was conducted during 2019–21. The ABY was implemented in a phased manner, and all the states did not implement ABY at the same time. In some states, the ABY was not launched even when data for NFHS-5 was collected. The NFHS-5 did not have a specific question for ABY, and hence it was not possible to segregate the ABY households. We would expect these to be added in the next round of NFHS.

## Conclusion

The paper provides comprehensive and robust estimates of OOPE and distress financing on cesarean deliveries in India. The findings of this study indicate that the rates of cesarean deliveries are quite high in India, and households incur high OOPE on them and, consequently, there is high distress financing for cesarean delivery. The inter-state and socioeconomic variations in OOPE and distress financing are high. Based on the findings, we recommend the following. First, there is a need to provide comprehensive pregnancy care that includes providing detailed information on the need and adverse consequences of cesarean deliveries to mothers during antenatal care and identifying high-risk pregnancies. This may help reduce cesarean deliveries and financial distress in India. Thus, monitoring/surveillance of pregnancy is recommended to reduce cesarean deliveries. Second, health is a state subject in India, and the majority of cesarean deliveries are conducted at private health centers. Thus regulating private health centers on the price of cesarean deliveries and auditing the price of cesarean deliveries by public authorities can reduce the OOPE and distress financing. Third, the Pradhan Mantri Ayushman Bharat Jan Arogya Yojana (PM-JAY), that has now been implemented across all states of India and union territories except Odisha, Delhi, and West Bengal, has great potential to reduce the high OOPE and distress financing. The PM-JAY, introduced in 2018, provides a cover of over Rs. 5 lakhs per family per year for secondary and tertiary care hospitalization across public and private empaneled hospitals in India. The program is in the implementation phase, and the inclusion of needy and poor households can reduce the high OOPE and distress financing. Along with the existing *Jannani Sishu Suraksha Karyakarma (JSSK)* under National Health Mission and state-specific schemes, the PM-JAY can reduce the high OOPE and distress financing on cesarean deliveries in India.

### Supplementary Information

**Additional file 1:** **Supplementary Table 1.** Percent distribution of cesarean and Non-cesarean delivery by type of health centers and background characteristics in, India, 2019-21. **Supplementary Table 2.** Mean OOPE in US$ for cesarean and non-cesarean delivery by type of health centers and background characteristics, India, 2019-21. **Supplementary Table 3.** OOPE on institutional delivery as a share of per capita state domestic product across the states of India, 2019-21. **Supplementary Table 4.** Percent distribution of source of financing and mean OOPE (in US$) institutional delivery in India. **Supplementary Table 5.** Percentage of births that incurred any distress financing and percent distribution of source of financing for cesarean delivery by background characteristics, India, 2019-21.

## Data Availability

Data for this study were extracted from the fifth round of the National family health survey of India 2019–2021, which is freely available in the public domain on the official website of DHS. https://dhsprogram.com/data/dataset/India_Standard-DHS_2020.cfm?flag=0

## Footnotes

1 *Source: Labour Bureau, Ministry of Labour and Employment, Government of India. Published in Reserve Bank of India Bulletin*.
