# Supplementary material for: Out of pocket expenditure and distress financing on cesarean delivery in India: evidence from NFHS-5
Source: BMC Health Serv Res. 2023 Sep 7;23:966. doi: 10.1186/s12913-023-09980-w (PMC10485997; doi:10.1186/s12913-023-09980-w)
Supplement: Supplementary file 1 — Additional file 1: Supplementary Table 1. Percent distribution of cesarean and Non-cesarean delivery by type of health centers and background characteristics in, India, 2019-21. Supplementary Table 2. Mean OOPE in US$ for cesarean and non-cesarean delivery by type of health centers and background characteristics, India, 2019-21. Supplementary Table 3. OOPE on institutional delivery as a share of per capita state domestic product across the states of India, 2019-21. Supplementary Table 4. Percent distribution of source of financing and mean OOPE (in US$) institutional delivery in India. Supplementary Table 5. Percentage of births that incurred any distress financing and percent distribution of source of financing for cesarean delivery by background characteristics, India, 2019-21. [file 12913_2023_9980_MOESM1_ESM.docx]

**Supplementary Tables:**

**Supplementary Table 1: Percent distribution of cesarean and Non-cesarean delivery by type of health centers and background characteristics in, India, 2019-21**

| **Background variables** | **Delivery at home** | **Private and Cesarean** | **Private and Non-Cesarean** | **Public and cesarean** | **Public and Non-cesarean** |
| --- | --- | --- | --- | --- | --- |
| **Mothers age** |  |  |  |  |  |
| 15-24 | 8.35 | 11.31 | 13.04 | 9.84 | 57.47 |
| 25-34 | 9.69 | 15.14 | 15.15 | 10.14 | 49.88 |
| 35+ | 14.78 | 16.39 | 14.39 | 9.28 | 45.16 |
| **Sex of child** |  |  |  |  |  |
| Male | 9.66 | 13.88 | 14.59 | 9.93 | 51.94 |
| Female | 9.8 | 14.17 | 14.16 | 10 | 51.86 |
| **Mothers education** |  |  |  |  |  |
| No education | 23.23 | 4.55 | 8.5 | 4.55 | 59.16 |
| Primary | 13.87 | 6.65 | 9.26 | 7.83 | 62.39 |
| Secondary | 7.4 | 11.94 | 12.56 | 11.95 | 56.15 |
| Higher secondary | 2.52 | 25.11 | 22.15 | 11.77 | 38.46 |
| **Birth order** |  |  |  |  |  |
| 1 | 4.21 | 19.21 | 16.23 | 12.75 | 47.6 |
| 2 | 7.56 | 15.3 | 14.39 | 11.73 | 51.03 |
| 3 | 14.02 | 8.41 | 13.45 | 5.98 | 58.14 |
| 4+ | 24.97 | 3.8 | 10.74 | 2.68 | 57.81 |
| **Residence** |  |  |  |  |  |
| Urban | 5.08 | 21.84 | 21.02 | 12.9 | 39.16 |
| Rural | 11.55 | 10.94 | 11.79 | 8.81 | 56.91 |
| **Wealth quintile** |  |  |  |  |  |
| Poorest | 21.99 | 3.8 | 5.34 | 4.91 | 63.96 |
| Poorer | 11.11 | 7.71 | 9.29 | 9.31 | 62.58 |
| Middle | 6.5 | 12.86 | 13.31 | 13.23 | 54.1 |
| Richer | 3.96 | 19.55 | 19.21 | 12.98 | 44.3 |
| Richest | 2 | 30.21 | 28.33 | 10.35 | 29.11 |
| **Mother BMI** |  |  |  |  |  |
| Underweight | 12.93 | 7.73 | 11.75 | 7.35 | 60.24 |
| Normal | 10.32 | 11.74 | 13.77 | 9.12 | 55.04 |
| Overweight | 5.31 | 25.32 | 17.55 | 15.02 | 36.8 |
| **Pregnancy complications** |  | |  |  |  |
| No | 15.56 | 12.53 | 14.64 | 7.57 | 49.7 |
| Yes | 7.5 | 14.58 | 14.3 | 10.88 | 52.74 |
| **Repeated Cesarean** |  |  |  |  |  |
| No | 10.1 | 12.34 | 14.9 | 8.74 | 53.91 |
| Yes | 1.16 | 52.08 | 2.9 | 37.62 | 6.25 |

**Supplementary Table 2: Mean OOPE in US$ for cesarean and non-cesarean delivery by type of health centers and background characteristics, India, 2019-21**

| **Background variables** | **Public and Non-cesarean** | **Public and cesarean** | **Private and Non-Cesarean** | **Private and Cesarean** |
| --- | --- | --- | --- | --- |
| **Mothers age** |  |  |  |  |
| 15-24 | 34 | 93 | 210 | 450 |
| 25-34 | 35 | 101 | 246 | 508 |
| 35+ | 36 | 113 | 281 | 530 |
| **Sex of child** |  |  |  |  |
| Male | 36 | 99 | 238 | 493 |
| Female | 34 | 100 | 239 | 498 |
| **Mothers education** |  |  |  |  |
| No education | 28 | 93 | 155 | 417 |
| Primary | 32 | 87 | 164 | 449 |
| Secondary | 36 | 97 | 211 | 456 |
| Higher secondary | 42 | 107 | 289 | 532 |
| **Birth order** |  |  |  |  |
| 1 | 39 | 104 | 265 | 512 |
| 2 | 36 | 92 | 241 | 484 |
| 3 | 31 | 104 | 201 | 474 |
| 4+ | 29 | 113 | 182 | 464 |
| **Residence** |  |  |  |  |
| urban | 40 | 94 | 276 | 532 |
| rural | 34 | 103 | 212 | 467 |
| **Wealth Index** |  |  |  |  |
| poorest | 29 | 97 | 152 | 379 |
| poorer | 34 | 103 | 171 | 429 |
| middle | 37 | 95 | 205 | 453 |
| richer | 39 | 101 | 234 | 500 |
| richest | 42 | 102 | 308 | 553 |
| **Mothers BMI** |  |  |  |  |
| underweight | 31 | 90 | 204 | 452 |
| Normal | 34 | 96 | 230 | 475 |
| Overweight | 42 | 107 | 271 | 531 |
| **Pregnancy complications** |  |  |  |  |
| No | 36 | 105 | 230 | 488 |
| Yes | 35 | 98 | 242 | 498 |
| **Repeated Cesarean** |  |  |  |  |
| No | 35 | 101 | 238 | 497 |
| Yes | 40 | 94 | 249 | 487 |

** 1 dollar = 73.78 INR* (*(average of 2019, 2020 and 2021 exchange prices as the survey was done during the period)*

**Supplementary Table 3: OOPE on institutional delivery as a share of per capita state domestic product across the states of India, 2019-21**

|  | **OOPE as a share of SDPP (in US$*)** | | | | |
| --- | --- | --- | --- | --- | --- |
| **State**** | **Private and Cesarean** | **Private and Non-Cesarean** | **Public and Cesarean** | **Public and Non-Cesarean** | **Total** |
| India | 27.03 | 13.02 | 5.39 | 1.91 | 7.19 |
| Andaman & Nicobar | 30.24 | 17.68 | 1.92 | 1.49 | 5.01 |
| Andhra Pradesh | 19.68 | 11.24 | 3.03 | 1.63 | 8.81 |
| Arunachal Pradesh | 33.53 | 15.25 | 12.39 | 5.10 | 5.99 |
| Assam | 56.33 | 22.41 | 17.10 | 6.04 | 10.88 |
| Bihar | 76.14 | 28.29 | 25.55 | 6.17 | 14.23 |
| Chandigarh | 16.33 | 13.78 | 2.86 | 1.15 | 3.39 |
| Chhattisgarh | 32.37 | 16.49 | 8.55 | 1.66 | 5.98 |
| Delhi | 14.04 | 8.54 | 1.73 | 0.70 | 3.95 |
| Goa | 11.25 | 8.30 | 1.31 | 0.89 | 4.86 |
| Gujarat | 17.11 | 6.16 | 2.75 | 0.74 | 5.60 |
| Haryana | 17.30 | 7.49 | 1.56 | 0.70 | 4.84 |
| Himachal Pradesh | 25.73 | 13.30 | 3.51 | 2.04 | 5.24 |
| Jammu & Kashmir | 33.54 | 19.30 | 8.48 | 5.60 | 7.80 |
| Jharkhand | 47.57 | 19.32 | 10.82 | 2.63 | 8.51 |
| Karnataka | 18.55 | 10.71 | 4.73 | 2.07 | 6.83 |
| Kerala | 24.15 | 15.35 | 4.04 | 2.86 | 13.52 |
| Madhya Pradesh | 45.41 | 21.62 | 7.18 | 1.61 | 5.81 |
| Maharashtra | 21.72 | 11.07 | 3.46 | 1.53 | 7.30 |
| Manipur | 79.43 | 37.84 | 40.28 | 19.95 | 28.85 |
| Meghalaya | 60.08 | 17.00 | 9.08 | 2.70 | 5.60 |
| Mizoram | 12.92 | 8.43 | 6.95 | 1.78 | 3.01 |
| Nagaland | 28.65 | 16.14 | 16.93 | 4.08 | 4.08 |
| Orissa | 32.12 | 14.69 | 10.63 | 3.40 | 7.67 |
| Puducherry | 16.92 | 12.18 | 1.87 | 2.05 | 4.85 |
| Punjab | 26.75 | 12.90 | 5.03 | 2.15 | 10.49 |
| Rajasthan | 30.99 | 12.41 | 5.37 | 1.89 | 5.01 |
| Sikkim | 7.97 | 3.61 | 3.24 | 1.59 | 2.64 |
| Tamil Nadu | 23.03 | 15.41 | 2.36 | 1.66 | 8.18 |
| Telangana | 14.80 | 8.52 | 2.64 | 1.84 | 7.58 |
| Tripura | 28.76 | 12.09 | 9.40 | 3.97 | 6.84 |
| Uttar Pradesh | 56.29 | 21.47 | 15.53 | 3.40 | 12.37 |
| Uttarakhand | 23.47 | 11.70 | 4.81 | 1.45 | 6.49 |
| West Bengal | 24.22 | 12.83 | 5.17 | 1.96 | 6.68 |

**Supplementary Table 4: Percent distribution of source of financing and mean OOPE (in US$) institutional delivery in India**

| **Source of Financing** | **Mean OOPE** | **95 % CI** | **N** |
| --- | --- | --- | --- |
| Only Savings | 157 | (156, 159) | 97,943 |
| Selling & borrowings | 176 | (171, 180) | 14,848 |
| Only Insurance | 151 | (143, 160) | 3,686 |
| Saving along with selling & borrowing | 302 | (296, 309) | 10,740 |
| Insurance along with selling & borrowing | 333 | (271, 395) | 136 |
| Insurance along with savings | 379 | (341, 417) | 463 |
| Savings, selling & borrowing and insurance | 539 | (446, 631) | 87 |
| Did not pay | 0 | (0, 0) | 48,940 |

**Supplementary Table 5: Percentage of births that incurred any distress financing and percent distribution of source of financing for cesarean delivery by background characteristics, India, 2019-21**

| **Background variables** | **Insurance & Others** | **Savings** | **Selling or Borrowing** | **Saving along with borrowing & selling** | **Didn't Pay** | **Any Distress financing** |
| --- | --- | --- | --- | --- | --- | --- |
| **Mothers age** |  |  |  |  |  |  |
| 15-24 | 4.64 | 60.35 | 12.56 | 12.78 | 9.67 | 25.34 |
| 25-34 | 4.02 | 65.83 | 10.16 | 11.79 | 8.20 | 21.95 |
| 35+ | 3.50 | 67.25 | 8.80 | 11.25 | 9.19 | 20.06 |
| **Sex of child** |  |  |  |  |  |  |
| male | 3.88 | 64.39 | 10.57 | 12.40 | 8.76 | 22.97 |
| female | 4.45 | 64.42 | 40.87 | 11.58 | 8.67 | 22.46 |
| **Mothers education** |  |  |  |  |  |  |
| No education | 3.68 | 51.69 | 16.53 | 17.68 | 10.42 | 34.21 |
| Primary | 5.15 | 53.77 | 12.70 | 17.62 | 10.76 | 30.32 |
| Secondary | 4.47 | 60.85 | 12.91 | 12.91 | 8.87 | 25.82 |
| Higher secondary | 3.83 | 70.64 | 7.83 | 9.65 | 8.04 | 17.49 |
| **Birth order** |  |  |  |  |  |  |
| 1 | 4.04 | 65.67 | 10.04 | 11.35 | 8.89 | 21.39 |
| 2 | 4.34 | 65.04 | 10.87 | 11.26 | 8.49 | 22.13 |
| 3 | 4.19 | 58.98 | 12.96 | 15.36 | 8.51 | 28.32 |
| 4+ | 3.07 | 55.73 | 11.32 | 20.25 | 9.63 | 31.57 |
| **Place of Residence** |  |  |  |  |  |  |
| Urban | 3.76 | 69.12 | 7.88 | 10.64 | 8.60 | 18.52 |
| Rural | 4.42 | 61.15 | 12.67 | 12.97 | 8.80 | 25.64 |
| **Wealth Index** |  |  |  |  |  |  |
| Poorest | 5.18 | 49.34 | 17.93 | 16.31 | 11.23 | 34.25 |
| Poorer | 5.06 | 53.04 | 16.51 | 15.44 | 9.96 | 31.95 |
| Middle | 4.40 | 61.68 | 12.72 | 12.28 | 8.93 | 24.99 |
| Richer | 4.12 | 66.77 | 9.71 | 11.67 | 7.73 | 21.38 |
| Richest | 3.24 | 74.29 | 5.16 | 9.20 | 8.10 | 14.37 |
| **Mothers BMI** |  |  |  |  |  |  |
| Underweight | 3.79 | 59.94 | 13.31 | 12.81 | 10.15 | 26.11 |
| Normal | 4.05 | 64.23 | 10.50 | 12.33 | 8.89 | 22.83 |
| Overweight | 4.37 | 66.07 | 10.52 | 11.26 | 7.77 | 21.79 |
| **Pregnancy complications** |  |  |  |  |  |  |
| No | 4.68 | 61.87 | 12.61 | 11.65 | 9.19 | 24.26 |
| Yes | 3.98 | 65.17 | 10.14 | 12.13 | 8.57 | 22.27 |
| **Repeated Caesarean** |  |  |  |  |  |  |
| No | 4.00 | 64.84 | 10.48 | 11.80 | 8.89 | 22.28 |
| Yes | 4.95 | 62.10 | 11.96 | 13.18 | 7.80 | 25.15 |
